# Skin-sparing mastectomy and immediate latissimus dorsi flap reconstruction: a retrospective analysis of the surgical and patient-reported outcomes

**DOI:** 10.1186/1477-7819-10-259

**Published:** 2012-11-29

**Authors:** Zisun Kim, Sang Gue Kang, Jung Ho Roh, Ji Hye Park, Jihyoun Lee, SungYong Kim, Cheol Wan Lim, Min Hyuk Lee

**Affiliations:** 1 Department of Surgery, Soonchunhyang University Hospital, Hannam-dong, Yongsan-gu, Seoul, 140-743, Korea; 2 Plastic and Reconstructive Surgery, Soonchunhyang University Hospital, Hannam-dong, Yongsan-gu, Seoul, 140-743, Korea

**Keywords:** Breast cancer, Breast neoplasm, Mastectomy, Latissimus dorsi flap, Breast reconstruction, Patient satisfaction

## Abstract

**Background:**

Skin-sparing mastectomy (SSM) and latissimus dorsi (LD) flap immediate breast reconstruction (IBR) is a tailored surgical procedure. The surgical and patient-reported outcome (PRO) of SSM and LD IBR were assessed.

**Methods:**

Retrospective data of 146 SSMs performed by a single surgeon was reviewed. Among patients included in the data, 65 patients underwent SSM and LD IBR without a prosthetic implant. A survey estimating the degree of patient satisfaction (poor, fair, good, and excellent) as regards the cosmetic outcomes of surgery was performed. The patients were divided into two groups according to their degree of satisfaction (excellent group versus non- excellent group), and analysis was done to identify factors affecting the highest patient satisfaction.

**Results:**

The mean age of the patients was 48.4 years, and pathological results were: infiltrating ductal carcinoma (n = 48, 73.8%), ductal carcinoma *in situ* (n = 15, 23.1%), and others (n = 2, 3.1%). One patient received postmastectomy radiotherapy. After a mean follow-up of 34 months, no local recurrence occurred. There was no skin necrosis or LD flap loss. Donor site morbidities were seroma (n = 8, 12.3%), scarring (n = 8, 12.3%), and back pain (n = 6, 9.2%). Fifty patients (76.9%) were satisfied and 40% reported their degree of satisfaction as excellent. Breast symmetry (*P* <0.001), nipple cosmesis (*P* <0.001), visual difference of bilateral breasts (*P* = 0.021), and panel assessment score (*P* <0.001) were factors that affected the highest patient satisfaction.

**Conclusions:**

Our SSM and LD IBR was safe, with no local recurrence and low morbidities, and produced a sufficiently high level of patient satisfaction. Achieving breast symmetry and nipple cosmesis would be the key to meeting the patient’s expectation.

## Background

The fundamental goal in surgical management of breast cancer is to achieve local control and to provide information for planning adjuvant local and systemic therapy. Over the past decades, surgical management of breast cancer has evolved from radical mastectomy to breast-conserving surgery. And most recently, the concept of surgical management is the pursuit of tailored breast surgery to achieve oncological safety and maximal aesthetic results together as the patient-reported outcome (PRO) has emerged as another fundamental goal.

Skin-sparing mastectomy (SSM), first introduced in 1991 [1,2], refers to a mastectomy which involves *en-bloc* removal of all breast tissue and nipple-areola complex while preserving the native breast skin and the infra-mammary fold. The adjacent biopsy scar, and skin overlying the superficial tumor could also be excised [3].

SSM followed by immediate breast reconstruction (IBR) with autologous tissue can be achieved utilizing options such as latissimus dorsi (LD) myocutaneous flap (with or without prosthetic implant) and transverse rectus abdominis myocutaneous (TRAM) flap. Immediate LD flap reconstruction without prosthetic implant is the most common sequence after SSM at our institution, since Korean breast cancer patients generally have small to moderately-sized breasts. Compared to delayed breast reconstruction, IBR is beneficial in relieving psychological trauma to the patient by restoring the breast mound after operation, and allowing fewer hospital admissions and operations with concomitant anesthesia. Also, the oncological safety of SSM with IBR has been demonstrated in the literature [3-8].

One of the most important issues after SSM and IBR, along with the oncological safety, might be the PRO of the surgical procedure. The aim of present study was to estimate the degree of patient satisfaction of SSM and LD IBR and surgical outcomes as regards to safety, post-operative complications/morbidities, and aesthetic results. To the best of our knowledge, this is one of the largest series of data for breast reconstruction after SSM, using LD flap without prosthetic implant, reported in the literature.

## Methods

### Patient selection

A total of 145 consecutive patients underwent SSM at our institution from March 2000 to March 2011. Patients with indications for mastectomy with no skin involvement were offered SSM. In our series, stage 0 to IIIA (Tis to T2, N0 to N2) breast cancer patients, according to the American Joint Committee on Cancer (AJCC) TNM staging system were included. All 145 patients received IBR after SSM, and one patient had bilateral SSM and IBR due to bilateral breast cancer. The standard operative procedures were performed by the most senior surgeon in cooperation with a plastic surgeon. A survey estimating the degree of patient satisfaction after surgery (*poor, fair, good,* and *excellent*) was performed at post-operative follow-ups (range, 1.6 to 89.9 months). Among 85 patients (58.6%) who were eligible for questionnaire survey, 65 patients (76.5%) had LD flap reconstruction, 11 (12.9%) had LD flap reconstruction with prosthetic implant, and 9 (10.6%) had TRAM flap reconstruction. Sixty-five patients who underwent SSM and LD IBR without prosthetic implant were included in the analysis. Written informed consent was obtained from the patients for publication of this report and any accompanying images.

### Surgical procedure

**Figure 1:**
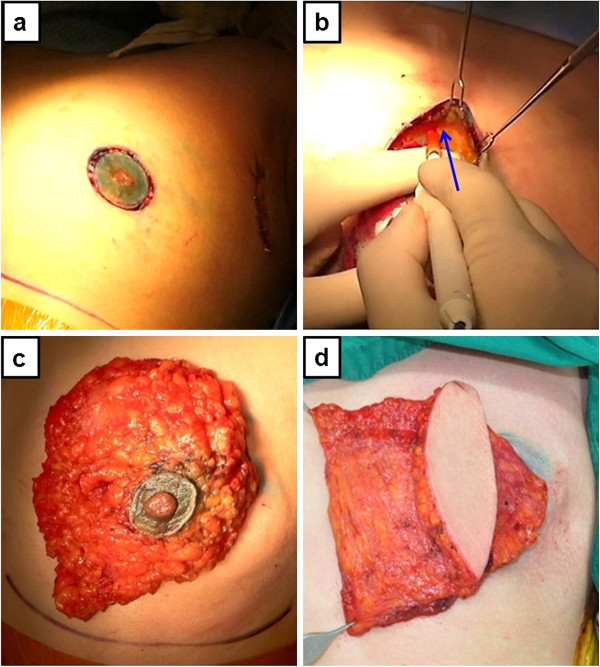
A 44 year-old woman diagnosed with stage IIA breast cancer of the left breast received skin-sparing mastectomy and immediate latissimus dorsi myocutaneous flap reconstruction: (a) circum-areolar skin incision, (b) dissection at the superficial layer of the superficial fascia, the blue arrow indicates dissection plane, (c) mastectomy specimen, (d) transferred latissimus dorsi flap through a subcutaneous tunnel.

SSM was performed using a circum-areolar incision (Figure 1a) in the majority of our cases, and overlying skin was excised when the tumor was close to the skin. The infra-mammary fold was preserved. Sentinel lymph node (SLN) biopsy was done through a separate axillary incision, and axillary lymph node dissection was performed when an intra-operative frozen section of the SLN showed presence of malignancy. Attention was paid to identifying and dissecting the superficial layer of the superficial fascia (Figure 1b, 1c). After SSM, the thoracodorsal vessels were identified, and the anterior portion of the LD muscle was identified and dissected through an anterior approach. Then the patient was repositioned in the lateral decubitus position for LD flap harvest, and skin incision was made at the back within the brassière line. The LD flap was elevated, rotated on the thoracodorsal neurovascular pedicle, and then was transferred subcutaneously to the mastectomy defect (Figure 1d). Closed suction drains were left at the LD donor site, mastectomy defect and axilla.

### Assessment of surgical outcomes

**Figure 2:**
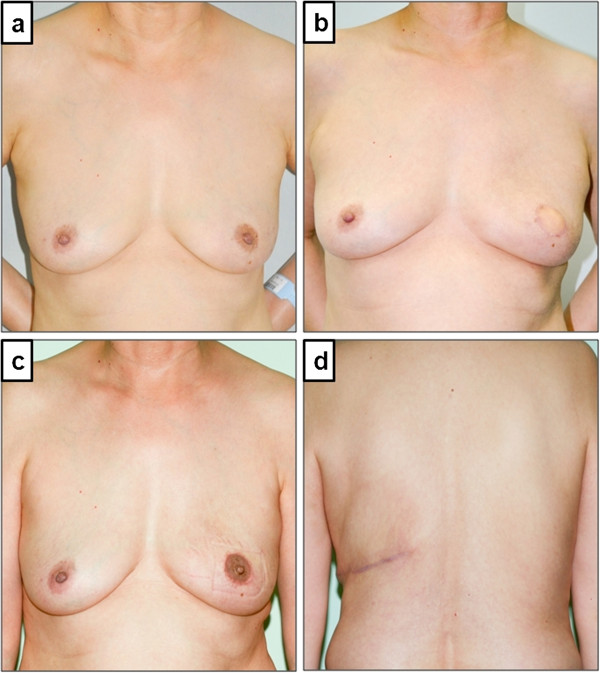
**A 56 year-old woman with ductal carcinoma *in situ *received left skin-sparing mastectomy and immediate latissimus dorsi myocutaneous flap reconstruction without an implant. **Satisfaction score of the patient was 10, which was considered *excellent* (**a**) preoperative state, (**b**) post-operative state without nipple reconstruction, (**c**) post-nipple reconstruction state, (**d**) back scar.

A patient survey was performed on follow-up visits at out-patient clinic. The degree of patient satisfaction regarding surgical outcomes was assessed using a questionnaire including an analogue scale ranged from 1 to 10 (Figure 2). None of the patients gave their score as 1 or 2, hence, the degree of patient satisfaction was determined as *poor* (score 3 to 4), *fair* (score 5 to 6), *good* (score 7 to 8), and *excellent* (score 9 to 10), according to the Harris cosmetic scale [9].

Surgical outcomes in terms of post-operative complications including hematoma, infection, scarring, dorsal seroma, skin necrosis, back pain, and aesthetic outcomes such as breast symmetry, visual difference of bilateral breasts, breast contour, and nipple cosmesis were assessed by a panel of two judges (operating surgeon and breast clinic nurse).

The patients were divided into two groups according to their degree of satisfaction. Group 1 was patients with *non-excellent* satisfaction (*poor*, *fair*, and *good*), and group 2 was patients with *excellent* satisfaction. The clinico-pathological characteristics, surgical outcomes, and aesthetic results were compared between the two groups. An analysis to determine factors affecting the highest patient satisfaction was performed.

### Data analysis

All statistical analyses were performed using SPSS software (SPSS, version 15.0, Chicago, IL, USA). Student’s *t-*test was used to compare the means of continuous variables, and chi-squared test and Fisher’s exact test were used for univariate comparison of categorical variables. Pearson’s simple correlation test was used to evaluate the inter-rater associations. All *P-*values were two-sided, and *P* <0.05 was considered significant.

## Results

### Patients and clinico-pathological characteristics

A total of 65 patients underwent SSM and LD IBR without prosthetic implant. The mean age of the patients was 48.4 years (range, 21 to 74), and pathological results were: infiltrating ductal carcinoma in 48 patients (73.8%), ductal carcinoma *in situ* in 15 (23.1%), and infiltrating lobular carcinoma in 2 (3.1%). The mean tumor size was 20 mm (range, 2 to 48 mm). One patient (1.5%) who had 4 axillary lymph node metastases (N2) received post mastectomy radiotherapy, and 29 patients (44.6%) received adjuvant chemotherapy including cyclophosphamide, methotraxate, and fluorouracil (n = 16); fluorouracil, doxorubicin, and cyclophosphamide (n = 4); doxorubicin, cyclophosphamide followed by docetaxel (n = 9). Fifteen patients (23.1%) received anti-hormonal therapy. After a mean follow-up period of 34 months (range, 1.6 to 89.9 months), none of the patients developed local recurrence. Only 1 patient (1.5%) developed an axillary metastasis, and the overall patient survival was 100%. The two patient groups were similar as regards to age, stage distribution, tumor histology, and clinical characteristics (Table 1).

**Table 1:** The clinico-pathologic characteristics of the study population

	**Group 1 (n = 39)**	**Group 2 (n = 26)**	***P*-value**
	**mean±SD (range)/n (%)**	**mean±SD (range)/n (%)**	
Age, years	49.64 ± 10.6 (21to 74)	46.6 ± 7.7 (33 to 64)	0.209
Follow-up, months	36.5 ± 26.3 (1.6 to 89.9)	30.4 ± 21.9 (1.6 to 84.1)	0.333
Pathology			0.446
Infiltrating ductal carcinoma	29 (74.4)	19 (73.1)	
Ductal carcinoma in situ	8 (20.5)	7 (26.9)	
Infiltrating lobular carcinoma	2 (5.1)	0	
Tumor size, cm	2.1 ± 0.9 (0.2 to 4.8)	1.8 ± 0.9 (0.2 to 3.5)	0.304
Stage			0.274
0	8 (20.5)	7 (26.9)	
I	16 (41.0)	13 (50.0)	
IIA	10 (25.6)	3 (11.5)	
IIB	4 (10.3)	3 (11.5)	
IIIA	1 (2.6)	0	
T			0.493
Tis	8 (20.5)	7 (26.9)	
T1	17 (43.6)	13 (50.0)	
T2	14 (35.9)	6 (23.1)	
N			0.489
N0	32 (82.0)	23 (88.5)	
N1	6 (15.4)	3 (11.5)	
N2	1 (2.6)	0	
Adjuvant chemotherapy			0.441
No	18 (46.2)	18 (69.2)	
CMF^a^	12 (30.8)	4 (15.4)	
AC-T^b^	3 (7.7)	1 (3.8)	
TAC^c^	3 (7.7)	2 (7.7)	
FAC^d^	3 (7.7)	1 (3.8)	
Anti-hormone therapy			0.548
No	8 (20.5)	7 (26.9)	
Yes	31 (79.5)	19 (73.1)	
Radiotherapy			0.411
No	38 (97.4)	26 (100)	
Yes	1 (2.6)	0	
Local recurrence			
No	39 (100)	26 (100)	
Yes	0	0	
Regional Metastasis			0.411
No	38 (97.4)	26 (100)	
Yes	1 (2.6)	0	

^a^CMF:cyclophosphamide, methotraxate, flurouracil; ^b^A, doxorubicin; C, cyclophosphamide; T, docetaxel; ^c^T, docetaxel, A, doxorubicin, C, cyclophosphamide; ^d^FAC: flurouracil, epirubicin, cyclophosphamide.

### Post-operative complications and morbidities

The post-operative complications and morbidities were assessed (Table 2). The incidence of hematoma, wound infection, and skin flap necrosis/loss was 0%, 0%, and 0%, respectively. Donor site morbidities occurred in 22 cases; dorsal seroma in eight (12.3%), marked scarring in eight (12.3%), and back pain in six (9.2%). Dorsal seroma was defined as any fluid collection requiring aspiration after surgery which was managed with repeated percutaneous aspiration or placement of a closed suction drain. The type and frequency of complications and morbidities did not differ significantly between the two groups.

**Table 2:** Post-operative complications and morbidities

	**Group 1 (n = 39)**	**Group 2 (n = 26)**	***P*-value**
Hematoma	0	0	
Wound infection	0	0	
Flap complication			
Skin necrosis	0	0	
Fat necrosis	0	0	
Flap loss	0	0	
Donor site morbidity			
Seroma	6 (15.4%)	2 (7.7%)	0.355
Scarring	4 (10.3%)	4 (15.4%)	0.538
Back pain	4 (10.3%)	2 (7.7%)	0.726

### Aesthetic results and patient satisfaction

Nipple reconstruction was performed in 30 patients (46.2%) using the trefoil local flap technique (Figure 2c). Three patients (4.6%) had contralateral reduction mammoplasty as a balancing procedure. Mean donor site scar length was 17 cm (range, 7 to 24) (Figure 2d).

**Figure 3:**
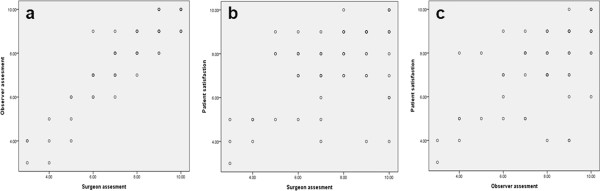
**Although the assessments of aesthetic results between surgeon and the breast clinic nurse showed a significant correlation (0.917, *P *<0.001, Figure **3a**), each result did not accord with the degree of patient satisfaction (Figure **3b**, Figure **3c**).**

A senior operating surgeon and the breast clinic nurse separately assessed the aesthetic outcomes as regards breast symmetry, visual difference of bilateral breasts, breast contour, and nipple cosmesis, and graded the score from 1 to 10, with 10 being the best. The mean score was 7.7 (range, 3 to 10) by the surgeon, and 7.9 (range, 3 to 10) by the breast clinic nurse. The mean score was 7.6 (range, 3 to 10) by the patients, which was considered *good*. Fifty patients (76.9%) were satisfied with their surgical outcome, and 26 (40%) gave their degree of satisfaction as *excellent* (score 9 to 10). No significant difference was seen in the type of skin incision, contralateral balancing procedure, nipple reconstruction, and donor site scar length between the two groups. As for the aesthetic results, breast size symmetry (*P* <0.001), visual difference of bilateral breasts (*P* = 0.021), nipple cosmesis (*P* <0.001), surgeon’s assessment (*P* <0.001), and breast clinic nurse’s assessment (*P* <0.001) were factors that significantly affected the *excellent* outcome for patient satisfaction (Table 3). Although the assessments of aesthetic outcome between the surgeon and the breast clinic nurse showed a significant correlation (0.917, *P* <0.001), their assessments did not accord with the degree of patient satisfaction (Figure 3).

**Table 3:** Cosmetic results

	**Group 1 (n = 39)**	**Group 2 (n = 26)**	***P*-value**
	**mean±SD (range)/n (%)**	**mean±SD (range)/n (%)**	
Nipple reconstruction			0.128
No	18 (46.2)	17 (65.4)	
Yes	21 (53.8)	9 (34.6)	
Contralateral procedure			0.334
No	38 (97.4)	24 (92.3)	
Yes (reduction)	1 (2.6)	2 (7.7)	
Donor site scar length, cm	17.0 ± 2.8 (7.0 to 24.0)	17.2 ± 1.8 (13.0 to 22.0)	0.788
Breast skin incision			0.241
Circum-areolar only	32 (82.1)	24 (92.3)	
Other incision added	7 (17.9)	2 (7.7)	
Breast size symmetry	6.9 ± 2.0 (3.0 to 10.0)	8.7 ± 1.4 (5.0 to 10.0)	<0.001
Visual difference			0.023
No	3 (7.7)	9 (34.6)	
−5	2 (5.1)	1 (3.8)	
−10	4 (10.3)	8 (30.8)	
−20	5 (12.8)	2 (7.7)	
−30	14 (35.9)	2 (7.7)	
−40	2 (5.1)	2 (7.7)	
−50	3 (7.7)	2 (7.7)	
−60	3 (7.7)	0	
−70	2 (5.1)	0	
+10	1 (2.6)	0	
Breast Contour			0.971
Good	31 (79.5)	21 (80.8)	
Depression	6 (15.4)	4 (15.4)	
Bulging	2 (5.1)	1 (3.8)	
Nipple cosmesis	7.7 ± 1.6 (3.0 to 10.0)	9.0 ± 0.9 (6.0 to10.0)	<0.001
Surgeon assessment	6.9 ± 2.1 (3.0 to 10.0)	8.8 ± 1.3 (5.0 to10.0)	<0.001
Breast clinic nurse assessment	7.2 ± 2.0 (3.0 to 10.0)	9.1 ± 1.0 (6.0 to 10.0)	<0.001

## Discussion

Our SSM and LD IBR without a prosthetic implant achieved a high level of patient satisfaction along with low complication rates. The procedure was safe, and none of our patients developed local recurrence.

SSM has been demonstrated as an oncologically safe procedure in patients with early stage breast cancer [10,11], and in selected patients with locally advanced breast cancer [5,12,13]. Local recurrence rate after SSM was reported as 3 to 12% [4]. Although concerns regarding local control and appropriate indications were raised [14], the available data do not support an increase in the risk of local recurrence with SSM when an accurate surgical dissection is performed [3,5,6]; and recently, in a meta-analysis of nine studies comprising 3,739 patients [4], no significant difference in local recurrence was noted between 1,104 patients with SSM and IBR, and 2,635 patients with conventional mastectomies without reconstruction. Most of our series included patients with early-stage breast cancer, and only one patient (1.5%) had locally advanced breast cancer. The local recurrence rate after a mean follow-up period of 34 months was 0%. Only one patient (1.5%) with initial stage IIA breast cancer developed an axillary metastasis and this occurred 84 months after operation. The patient received docetaxel and doxorubicin-based chemotherapy followed by radiation therapy, and remained safe without any evidence of disease progression at current follow-up.

Post-operative complications could compromise the aesthetic outcomes as well as the patient satisfaction. A recognized complication after SSM is skin flap necrosis reported to occur in 11% [15]. However, there was no case of skin flap necrosis or flap loss in our series. Accurate dissection of the superficial layer of the superficial fascia could have contributed to the enhanced surgical outcomes.

IBR after SSM has a virtue of producing pleasing aesthetic results. Since the breast skin is maximally preserved, IBR using the native skin envelope could be performed to achieve an optimal aesthetic result through a single-stage procedure. IBR can reduce the need for a contralateral balancing procedure to achieve breast symmetry [3,16]. Likewise, only three patients (4.6%) in our series received a contralateral balancing procedure. Alongside the superior aesthetic outcomes, IBR showed reduced psychological trauma to the patient, convenience of the operation, cost benefit, and patient safety [17].

Furthermore, IBR was reported to be oncologically safe [3-8], and not to result in delay or interference with the initiation of adjuvant chemotherapy [18]. A study of 166 patients by Caffo *et al.*[19] reported no marked increase in the rate of surgical complications due to adjuvant chemotherapy. Also in our series, none of the patients had to delay adjuvant chemotherapy after LD IBR.

Autologous LD myocutaneous flap has become a popular option for breast reconstruction since its introduction in the late 1970s [20,21]. Almost any patient could be a potential candidate for LD flap due to its robust blood supply [22,23], and ischemic complications after LD flap reconstruction are lower compared to other types of autologous flap reconstruction. Moreover, LD flap has produced a high level of patient satisfaction in a wide range of breast operations, from quadrantectomy to SSM or nipple-sparing mastectomy [24-26].

Rosson and colleagues [27] identified patients with small to moderately-sized breasts, inadequate amounts of abdominal tissue, or a history of previous abdominal surgery as ideal candidates for LD flap reconstruction. For most Korean women with a low to normal body mass index and small to moderately-sized breasts, LD flap could provide sufficient volume for breast reconstruction. And since a high proportion of Korean breast cancer patients are at child-bearing age [28], LD flap is an attractive option for Korean breast cancer patients.

A drawback of LD flap reconstruction is frequent formation of seroma at LD donor site, reported as 12 to 21% [29,30]. Dorsal seroma was managed with a prolonged suction drainage or repeated percutaneous aspiration at the outpatient clinic. Dorsal seroma occurred in eight patients (12.3%) in our series, which was lower than other reports. The lower morbidity rate, however, was not significantly related to higher patient satisfaction.

PRO of breast reconstruction has become increasingly important in clinical research. Although traditional surgical outcomes focused on morbidity and mortality as important measure, they are no longer sufficient on their own. Patient satisfaction and quality of life has become a crucial concern. In the present study, 50 patients (76.9%) were satisfied (*good* and *excellent*) with the surgical outcomes, and 40% reported their degree of satisfaction as *excellent* (score 9 to 10), demonstrating SSM and LD IBR without an implant could produce sufficiently satisfactory results. However, contrary to our expectations, the type of skin incision, breast contour, and donor site scar length did not significantly contribute to *excellent* patient satisfaction. The breast size symmetry (*P* <0.001), visual difference of bilateral breasts (*P* = 0.021), and the nipple cosmesis (*P* <0.001) related to the highest patient satisfaction, and 53.8% of patients who did not report their degree of satisfaction as *excellent*, cited asymmetry as one of the main reasons. Studies [24,31,32] suggested that achievement of breast symmetry was the main factor for patient aesthetic satisfaction after breast reconstruction. We fully agree and again have confirmed the importance of achieving breast symmetry in our series.

Thirty-five patients (53.8%) did not receive nipple reconstruction. The reasons for not doing nipple reconstruction were: fear of the second operation (n = 16, 45.7%); lack of necessity of the nipple (n = 7, 20%); premature time for nipple reconstruction (n = 8, 22.8%); and others (n = 4, 11.4%). Nipple cosmesis was the factor that significantly related to the highest patient satisfaction (*P* <0.001). Taken together, these results suggest that immediate nipple reconstruction at the time of IBR could be another potential factor that possibly enhances patient satisfaction.

Assessment by the surgeon (*P* <0.001) and the breast clinic nurse (*P* <0.001) were factors affecting the highest patient satisfaction, and the average score among the three groups (surgeon, breast clinic nurse, and patient) showed similar results (7.7, 7.9, and 7.6). The assessment between surgeon and breast clinic nurse showed a significant correlation (0.917, *P* <0.001), however, each of them did not accord with the degree of patient satisfaction (Figure 3). The authors could not identify confounding factors to explain the reason. Probably, minor discrepancies could have existed between patient’s subjective satisfaction and objective assessment by a third party assessor. Because expression of patient satisfaction is related both to the patient’s expectation and the surgical outcomes after breast reconstruction, patient-reported satisfaction could not be fully explained by only considering objective assessment measures made by others.

A limitation of the study was the assessment of patient satisfaction regarding surgical and aesthetic outcomes, which was measured only by an analogue scale, and hence the measure used to assess patient quality of life was insufficient. A structured questionnaire including subscales and long term satisfaction might be needed to more objectively assess patient satisfaction. It is also acknowledged that this study is limited by its small sample size and retrospective study design. Also, we believe that a uniform period of assessment after operation, and serial long term follow-up of patient satisfaction data will be able to provide more reliable results. The results of this study, however, highlight low rates of surgical complication as well as high degree of patient satisfaction following SSM and LD IBR without a prosthetic implant. Larger standardized measures focusing on improving PRO as related to aesthetic outcomes are needed in the future.

## Conclusions

The present study demonstrated that our SSM and LD IBR was safe with no local recurrence, and was associated with a high level of patient satisfaction. LD flap per se, without a prosthetic implant, could provide enough volume for breast reconstruction after SSM, with low rates of morbidities. In circumstances that postmastectomy radiotherapy is not expected to be required, accurate surgical technique and careful patient selection could provide a high level of patient satisfaction and superior aesthetic results after breast reconstruction.

## Abbreviations

AJCC: American Joint Committee on Cancer; IBR: Immediate breast reconstruction; LD: Latissimus dorsi; PRO: Patient-reported outcome; SLN: Sentinel lymph node; SSM: Skin- sparing mastectomy; TRAM: Transverse rectus abdominis myocutaneous.

## Competing interests

The authors declare that they have no competing interests.

## Authors’ contribution

Z Kim contributed to study conception and design, analysis of data, drafting of the manuscript and critical revision. SG Kang was responsible for analysis and interpretation of data. JH Roh was responsible for acquisition of data. JH Park was responsible for acquisition of data. J Lee was responsible for acquisition of data. SY Kim was responsible for interpretation of data. CW Lim was responsible for interpretation of data. MH Lee contributed to study conception and design and critical revision. All authors read and approved the final manuscript.
